# Vaginal and endometrial microbiota dysbiosis in patients with chronic endometritis: a systematic review and meta-analysis

**DOI:** 10.3389/fcimb.2026.1754297

**Published:** 2026-02-20

**Authors:** Ruiying Wang, Qi Cao, Xinyu Qiao, Yuchan Zhong, Wenjie Bo, Xin Huang, Yujing Li, Wei Huang

**Affiliations:** 1 Department of Obstetrics and Gynecology, West China Second University Hospital of Sichuan University, Chengdu, China; 2 Key Laboratory of Birth Defects and Related Diseases of Women and Children, Ministry of Education, Chengdu, China; 3 Key Laboratory of Chronobiology of National Health Commission of Sichuan University, Chengdu, China

**Keywords:** 16S rRNA sequencing, chronic endometritis, dysbiosis, endometrial microbiota, meta-analysis, vaginal microbiota

## Abstract

**Background:**

Chronic endometritis (CE), a persistent inflammatory condition of the endometrial lining, is clinically linked with adverse reproductive outcomes. It is currently hypothesized to be associated with infection, and is often treated with broad-spectrum antibiotics. However, the specific microbial alterations remain poorly defined due to heterogeneous findings.

**Methods:**

PubMed, Web of Science, Medline, Embase, Cochrane Library, and Scopus were searched for studies published up to July 2025. Studies were included if they compared CE patients to non-CE controls and analyzed vaginal or endometrial microbiota. Standardized mean difference (SMD) for alpha-diversity, odds ratio (OR) for microbial detection rates, with 95% confidence intervals (CIs) were calculated. Qualitative syntheses of beta-diversity and microbial abundance profiles were also performed.

**Result:**

Twenty-two studies (n = 1274 CE patients, n = 1109 controls) were included. Alpha-diversity indices showed no significant differences for both vaginal and endometrial microbiota. However, a subgroup analysis revealed a significant upregulation in endometrial Chao1 indices in 16S V4 sequencing studies (SMD = 0.38, 95% CI: 0.06 to 0.70, *I*^2^ = 0). Beta-diversity findings were inconsistent, though three endometrial studies reported significant intergroup differences. Qualitative synthesis revealed a decrease in *Lactobacillus* and an increase in opportunistic pathogens, including *Gardnerella* and *Sphingomonas*. Pooled analysis of microbial detection rates showed significantly higher prevalence for *Enterococcus* (OR = 4.93, 95% CI: 2.13 to 11.39, *I*^2^ = 48%) and *Ureaplasma* (OR = 6.30, 95% CI: 2.53 to 15.68, *I*^2^ = 0%) in CE patients.

**Conclusion:**

CE is associated with dysbiosis of the vaginal and endometrial microbiota, characterized by a shift from beneficial commensals to pathogenic microbes. This dysbiosis may contribute to an altered the intrauterine immune microenvironment.

**Systematic review registration:**

PROSPERO https://www.crd.york.ac.uk/PROSPERO/view/CRD420251115587, identifier CRD420251115587.

## Introduction

Chronic endometritis (CE) is an inflammatory state of the endometrial lining, characterized by plasma cell infiltration in the endometrial stroma (Buzzaccarini et al., 2020). CE is often asymptomatic or accompanied by mild symptoms, including pelvic pain, abnormal uterine bleeding (AUB), and so on (Greenwood and Moran, 1981). Although it is often clinically silent, accumulating evidence has demonstrated its significant potential association with recurrent implantation failure (RIF), recurrent pregnancy loss (RPL), and infertility (Bouet et al., 2016; Liu et al., 2018; McQueen et al., 2015). The precise etiology of CE remains unclear. Previous studies have identified potential associations with specific bacterial colonization in the reproductive tract, supporting the infection hypothesis (Buzzaccarini et al., 2020). However, this hypothesis is challenged by a subset of patients who do not respond to antibiotic therapy and those in whom no specific pathogens can be detected by traditional culture methods (Xiong et al., 2021). Meanwhile, as low-abundance pathogens prove challenging to culture, research has increasingly focused on microbial communities. With advancements in sequencing technologies, 16S ribosomal RNA (rRNA) gene sequencing has enabled a more comprehensive characterization of the reproductive tract microbiota and its potential role in CE pathogenesis (Clarridge, 2004).

The human reproductive tract constitutes a dynamic microbial continuum, exhibiting gradual compositional changes from the vagina to the endometrium, with a decrease in *Lactobacillus* along this tract (Chen et al., 2017). The vaginal microbiota is predominantly dominated by *Lactobacillus*, which maintain a protective acidic environment. A dysbiotic vaginal microbiota, marked by reduced *Lactobacillus* abundance and increased diversity, has been associated with adverse reproductive events (Smith and Ravel, 2017). Recent reviews or reports have highlighted that alterations in the vaginal or endometrial microbiota play a significant role in a broad spectrum of gynecological diseases, including uterine fibroids (Chen et al., 2017), adenomyosis (Zheng et al., 2025), endometriosis (Colonetti et al., 2023), and most notably, endometrial cancer (Aquino et al., 2024). The endometrial microbiota dysbiosis may also alter key inflammatory pathways crucial for successful embryo implantation and pregnancy (Benner et al., 2018).

Previous investigations have explored the relationship between CE and microbial alterations, but their findings have been inconsistent due to small sample sizes, heterogeneous methodologies, and varied diagnostic criteria. We conducted this systematic review and meta-analysis to synthesize the current evidence, identify consistent patterns of CE-associated dysbiosis, provide critical insights into the microbial etiology of CE, and guide future research for pathogenic mechanisms and inform therapeutic development for CE.

## Methods

This systematic review was preregistered with PROSPERO (CRD420251115587) and conducted according to the Preferred Reporting Items for Systematic Reviews and Meta-Analyses (PRISMA) reporting guideline (Moher et al., 2009).

### Search strategy

We conducted a comprehensive search across PubMed, Embase, Web of Science, and Cochrane Library for articles published up to July 2025, using a combination of terms related to chronic endometritis (“chronic endometritis” OR endometritis OR “endometrial inflammation”), microbiota (microbiome OR microbiota OR microflora OR bacteria OR dysbiosis OR “microbial community”), and anatomical sites (vaginal OR cervical OR endometrial OR uterine OR “reproductive tract”). The search was limited to original human studies, with no language restriction.

### Selection criteria

Two independent reviewers (WRY and CQ) screened titles and abstracts to identify potentially relevant studies. Subsequently, the full-text articles were assessed for final inclusion. Eligible studies met the following criteria: (1) applied an observational case-control design; (2) included reproductive-age women with confirmed CE and non-CE (NCE) controls; and (3) assessed vaginal or endometrial microbiota (including diversity, abundance, or microbial detection rates of specific agents). We excluded reports involving patients with active infections or recent use of antibiotics or probiotics before sampling. Disagreement was resolved by consulting a third reviewer (QXY).

### Data extraction

Data were extracted by two authors (ZYC and BWJ) and cross-checked by HX and LYJ. The following variables were extracted: study characteristics (first author, year, sample type, patient source, diagnostic criteria of CE, microbiome assessment method), group information (sample number, age), microbial diversity (alpha- and beta-diversity), taxonomic profiles at the phylum and genus levels, and microbial detection rate. Numerical data from graphs were extracted using WebPlotDigitizer (v.4.8) when necessary. Medians and inter-quartile ranges were transformed to means (M) and standard deviations (SD) using two web-based tools (https://www.math.hkbu.edu.hk/~tongt/papers/median2mean.html) (https://smcgrath.shinyapps.io/estmeansd).

### Risk of bias assessment

The quality of each included studies was assessed using the Joanna Briggs Institute Critical Appraisal Checklist for Case-Control Studies.

### Data analysis

For studies utilizing 16S rRNA sequencing, we performed meta-analysis for alpha-diversity (richness and evenness), and summarized the findings for beta-diversity (compositional differences) and microbial taxonomic abundance. For studies that analyzed microbial detection rates based different methods, we conducted a separate meta-analysis.

The meta-analysis for differences in alpha-diversity between CE patients and controls utilized the random-effects or fixed-effects model on standardized mean difference (SMD). Inter-study heterogeneity was assessed using the Restricted Maximum Likelihood and reported with the *I*^2^ statistic and its associated *p*-value. Significant heterogeneity was defined as *I*^2^ ≥ 50% or *p* < 0.05. Pooled results and 95% CIs were calculated with a random-effects model when significant heterogeneity was observed. Given that prior research suggests sample type, menstrual cycle, and diagnostic criteria may affect bacterial composition (Chen et al., 2017; Liu et al., 2024; Lüll et al., 2022), subgroup analyses and meta-regression were performed to explore sources of heterogeneity, stratified by patient source, sample type, age, menstrual cycle phase, diagnostic criteria, and 16S rRNA region. CE diagnoses based on immunohistochemistry (IHC) revealing ≥ 5 plasma cells per 10 high-power fields (HPF) were considered strongly positive. Sensitivity analysis was conducted by removing the high-risk studies.

For beta-diversity, we summarized and described the findings from each included study.

For the microbial taxonomic abundance, we focused on taxa reported as altered in two or more sequencing studies at the phylum and genus levels, considering the high heterogeneity and varied reporting methods. We characterized the variations (decreased, increased, or not changed) between women with CE and controls. The overall findings across studies were summarized in a “Total” row. Findings were considered potentially associated with CE only if consistently reported by at least two independent studies.

For microbial detection rates, meta-analysis was performed for species reported in three or more studies, using the odds ratio (OR) with a 95% CI to assess the association between the specific microbe and CE. The inter-study heterogeneity for microbial detection rates was assessed using the same methods as described above. Subgroup analyses and meta-regression were stratified by detective method and diagnostic criteria.

Publication bias was evaluated with funnel plots. Due to the limited number of studies, statistical tests for publication bias were not performed.

All analyses were conducted using the R software (4.2.2), with the “meta” package utilized for the meta-analysis. *P* < 0.05 was defined as statistically significant.

## Result

### Search results

**Figure 1:**
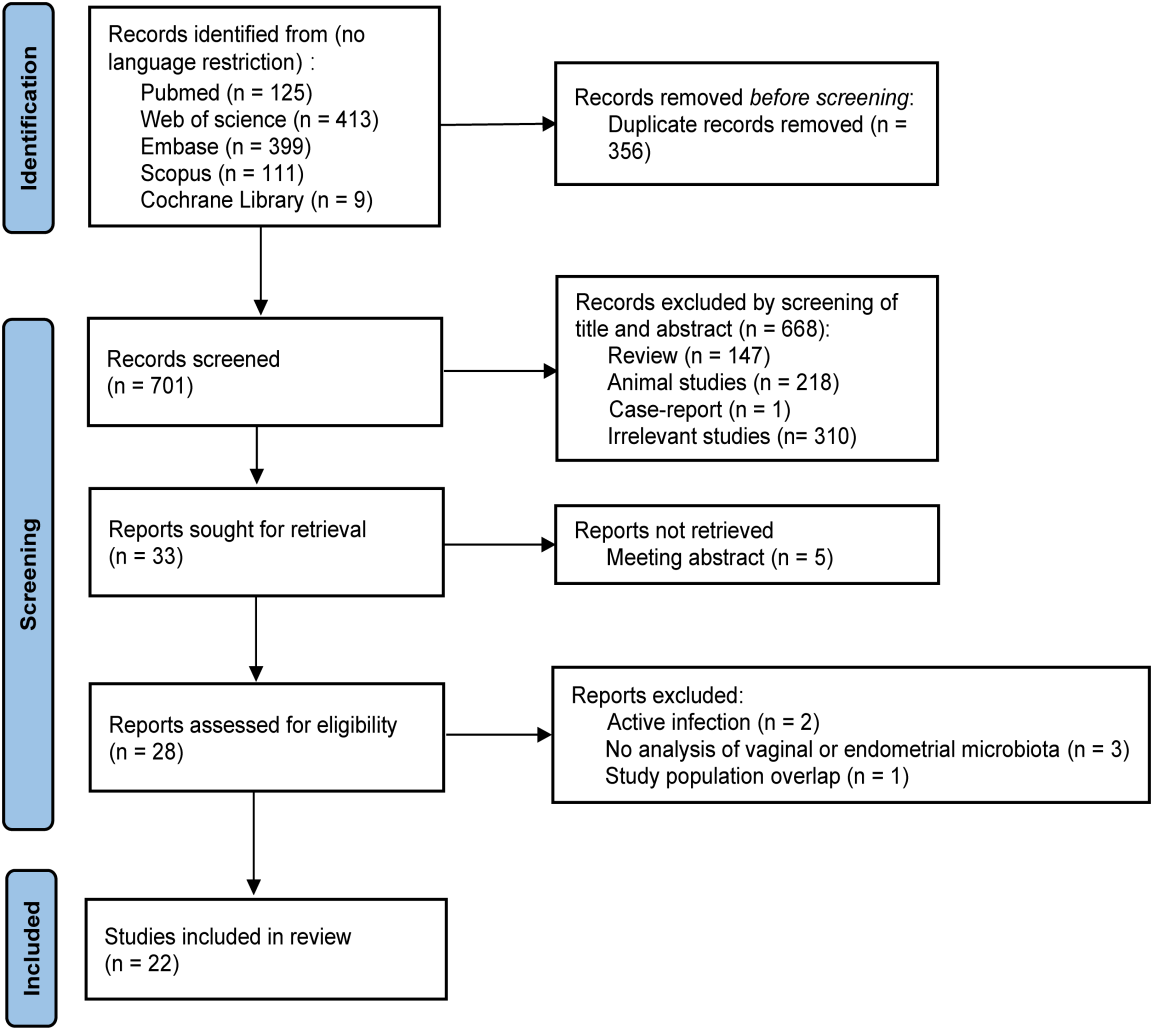
Flow diagram of the study selection.

Following the PRISMA search flowcharts, the search yielded 1057 published articles from PubMed, Embase, Scopus, Web of Science, and the Cochrane Library. Finally, we included a total of 22 original studies (Chen et al., 2021, 2023, 2021; Cicinelli et al., 2008; Danusevich et al., 2017; Fang et al., 2016; Han et al., 2024; Hiratsuka et al., 2025; Kobaidze and Padrul, 2017; Liang et al., 2023; Liu et al., 2024, 2019; Lozano et al., 2021; Lüll et al., 2022; Lyzikova, 2023; Muravyova et al., 2015; Sánchez-Ruiz et al., 2024; Takimoto et al., 2023; Tanaka et al., 2022; Tapilskaya et al., 2020; Voroshilina et al., 2020; Zhang et al., 2024). **Figure 1** presents the flowchart of the study process.

### Characteristics of included studies

These studies provided 22 case-control comparisons capturing 1274 CE patients and 1109 controls. The characteristics of the included studies are summarized in **Table 1**, stratified by the microbial assessment method. The majority of studies (12, 54.55%) were conducted in East Asia (China, Japan), with other studies conducted in Russia (n=6), Spain (n=2), Belarus (n=1), and Italy (n=1). Seventeen studies investigated the endometrial microbiota (via biopsy/fluid sampling), while five analyzed the vaginal microbiota (via swab sampling). The sample size of CE patients ranged between 10 and 438, but most studies (17, 77.27%) included fewer than 50 participants. Fifteen studies diagnosed CE based on the number of CD138-positive plasma cells by IHC, three studies used histological diagnosis via hematoxylin and eosin (HE) staining, one study relied on hysteroscopic findings, one study considered any of the above three methods positive, and 2 studies failed to document the diagnostic protocols for CE. For assessment of the microbiota, most studies (13, 59.09%) utilized 16S rRNA sequencing, one study utilized sequencing technology (details not provided), 3 studies (13.64%) used quantitative polymerase chain reaction, and 5 studies (22.73%) employed microbial culture methods. Excluding the study by Chen et al (Chen et al., 2021), all studies that assessed the microbiota using high-throughput sequencing technology utilized the IHC method for CE diagnosis.

**Table 1:** Characteristics of included studies.

Author/year	Country	Case		Control		Patients source	Sample type	Phases of the menstrual cycle	Diagnostic criteria for chronic endometritis	Microbial assessment methods	Diversity assessments
		Sample size	Age	Sample size	Age						
Studies utilizing 16S rRNA sequencing for microbial assessment											
Chen et al., 2021	China	14	Not reported	29	Not reported	RIF	ET	Secretory	Not reported	16S V4	α: Shannon
Chen et al., 2021	China	18	30.16± 0.006	45	30.51 ± 0.529	Infertility	EF	Secretory	>= 1 plasma cells/HPF via IHC	16S V4	α: Chao1, Shannon,
Chen et al., 2023	China	29	32.11 ± 4.46	42	32.33 ± 3.66	Infertility	EF	Proliferative	>= 4 plasma cells/HPF via IHC	16S V4	α: Shannon β
Fang et al., 2016	China	10	35.2 ± 1.83	10	30.9 ± 1.56	Women with endometrial polyps and healthy controls	VS, ET	Proliferative	>= 5 plasma cells/10 HPF via IHC	16S V4	α: Chao1, Shannon, Simpson β
Han et al., 2024	China	49	33.5 ± 3.8	49	33.7 ± 3.8	Infertility	VS	Proliferative	>= 5 plasma cells/30 HPF via IHC	16S V4	α: Chao1, Shannon β
Hiratsuka et al., 2025	Japan	36	35.7 ± 3.7	37	35.7 ± 3.7	Women with two failed embryo transfers	EF	Secretory	>= 1 plasma cells/10 HPF via IHC	Sequencing (details not provided)	NA
Liang et al., 2023	China	29	33 ± 7.6	85	33 ± 7.6	Infertility	ET	Proliferative	>= 1 plasma cells via IHC	16S V4	α: Chao1, Shannon β
Liu et al., 2019	China	12	35(34-39)	118	36(34-38)	Infertility	EF	Secretory	>5.15/10 mm2	16S V4	NA
Liu et al., 2024	China	22	31.55 ± 4.50	59	32.03 ± 2.88	Infertility women with previous IVF procedure failures	ET	Not reported	>= 1 plasma cells via IHC	16S V3-V4	α: Chao1, Shannon, Simpson, Ace β
Lozano et al., 2021	Spain	30	39.2	24	39.2	Women underwent transfer of frozen euploid embryos	VS, ET	Secretory	>= 2 plasma cells/5 HPF via IHC	16S V3-V4	α: Chao1, Shannon, Simpson β
Lüll et al., 2022	Russia	12	36.28 ± 4.51	11	36.28 ± 4.51	Women with previous IVF procedure failures	EF, ET	Secretory	>= 1 plasma cells via IHC	16S V3-V4	NA
Tanaka et al., 2022	Japan	20	38.1 ± 3.7	103	38.4 ± 4.1	Infertility	VS, EF	Secretory	>= ESPC density index 0.25	16S V4	α: Chao1, Shannon, PD β
Takimoto et al., 2023	Japan	11	37.07 ± 4.64	69	37.07 ± 4.64	RIF/PRL/fertile	ET	Secretory phase	>5.15/10 mm2 via IHC	16S V4	NA
Zhang et al., 2024	China	40	30.57 ± 2.64	40	32.88 ± 2.03	Asymptomatic women with RIF	EF	Secretory	>= 4 plasma cells/HPF via IHC	16S V3-V4	α: Chao1, Shannon β
Studies utilizing specific culture/PCR for microbial assessment											
Cicinelli et al., 2008	Italy	438	35.7 ± 8.2	100	36.3 ± 8.3	Women for diagnostic hysteroscopy	VS, ET	Proliferative	Hysteroscopic findings	Culture	NA
Danusevich et al., 2017	Russia	50	30.5 ± 0.6	50	30.2 ± 0.7	Infertility	Endometrial specimen	Not reported	Histological diagnosis via HE	Culture	NA
Kobaidze et al., 2017	Russia	42	34.11 ± 4.79	33	30.90 ± 7.09	Reproductive-age women	ET	Proliferative	Not reported	Culture	NA
Lyzikova, 2023	Belarus	230	Not reported	110	Not reported	Reproductive-age women	VS	Not reported	IHC (details not provided)	PCR	NA
Muravyova et al., 2015	Russia	38	31.8 ± 2.3	19	31.8 ± 2.3	Reproductive-age women with AUB/infertility	ET	Proliferative	Histological diagnosis via HE	Culture	NA
Sánchez-Ruiz et al., 2024	Spain	56	34.88 ± 4.62	54	35.33 ± 3.83	Infertility/ART	ET	Proliferative	hysteroscopy, pathology, and/or >= 5 plasma cells via IHC	Culture+PCR	NA
Tapilskaya et al., 2020	Russia	113	35(31-38)	32	35(31-38)	Infertility	ET	Proliferative/Secretory	>= 5 plasma cells/10 HPF via IHC	PCR	NA
Voroshilina et al., 2020	Russia	23	33 ± 5.2	19	33 ± 5.2	Reproductive-age women	ET	Proliferative	Histological diagnosis via HE	PCR	NA

### Risk of bias assessment

The risk of bias in each included study was shown in **Supplementary Table S1**. Nine studies had a high risk of bias, primarily due to the lack on reporting confounding factors and adjustment methods.

### Alpha-diversity

Ten studies provided precise data or statistical plots of alpha diversity.

**Figure 2:**
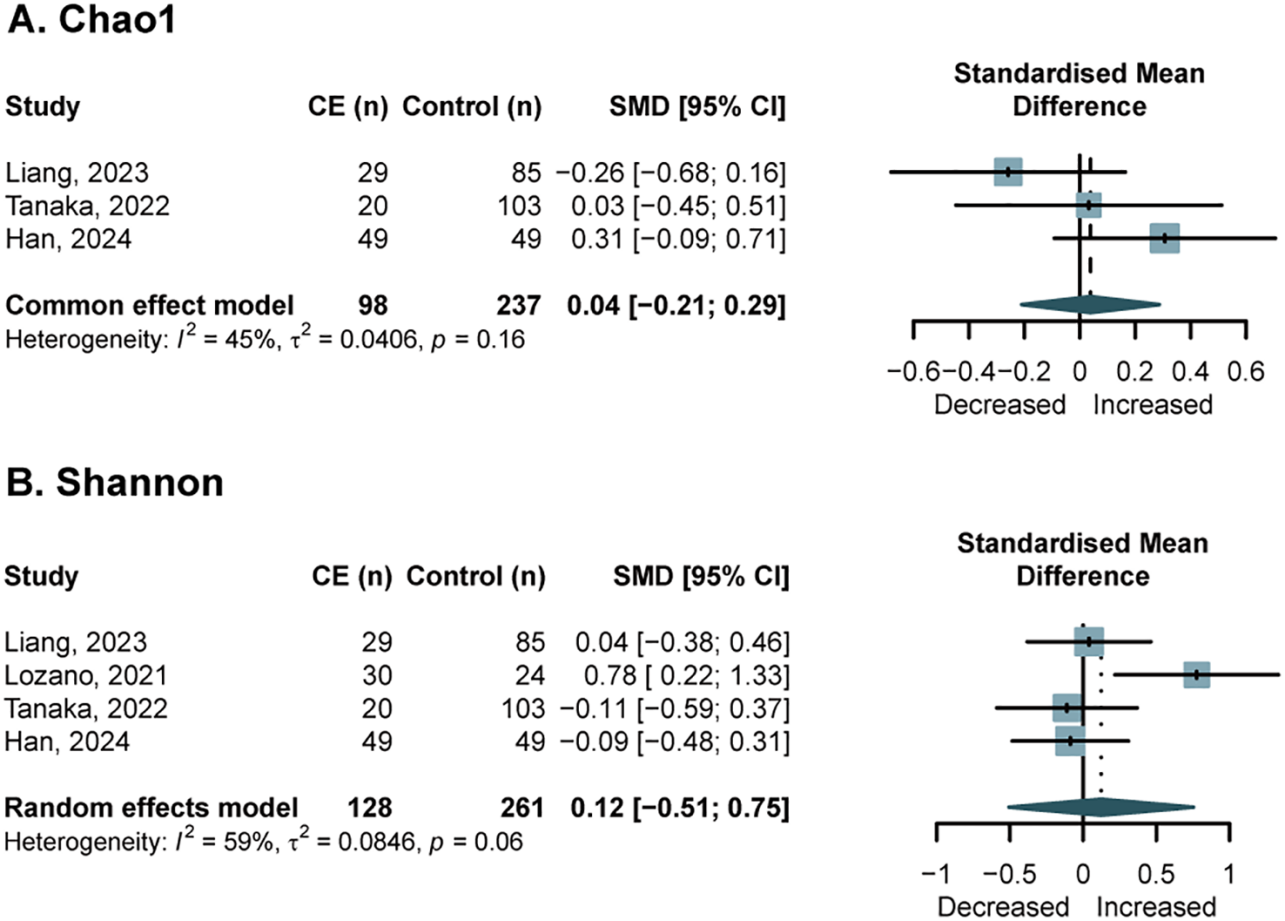
Forest plots of alpha-diversity in the vaginal microbiota of patients with CE compared with NCE controls. **(A)** Chao1; **(B)** Shannon index.

Regarding vaginal microbiota, three studies (Han et al., 2024; Liang et al., 2023; Tanaka et al., 2022) provided data on Chao1 in 98 CE and 237 controls, and four studies (Han et al., 2024; Liang et al., 2023; Lozano et al., 2021; Tanaka et al., 2022) provided Shannon data in 128 CE and 261 controls. No significant differences were found for Chao1 (SMD = 0.04, 95% CI, -0.21 to 0.29; *I*^2^ = 45%, *p* = 0.16) (**Figure 2A**) and Shannon indices (SMD = 0.12; 95% CI = -0.51 to 0.75; *I*^2^ = 59%, *p* = 0.06) (**Figure 2B**). The Simpson index was reported in two studies but could not be pooled due to incomplete data (Fang et al., 2016; Lozano et al., 2021), while phylogenetic diversity was only reported in one study, and no significant intergroup differences were found (Tanaka et al., 2022).

**Figure 3:**
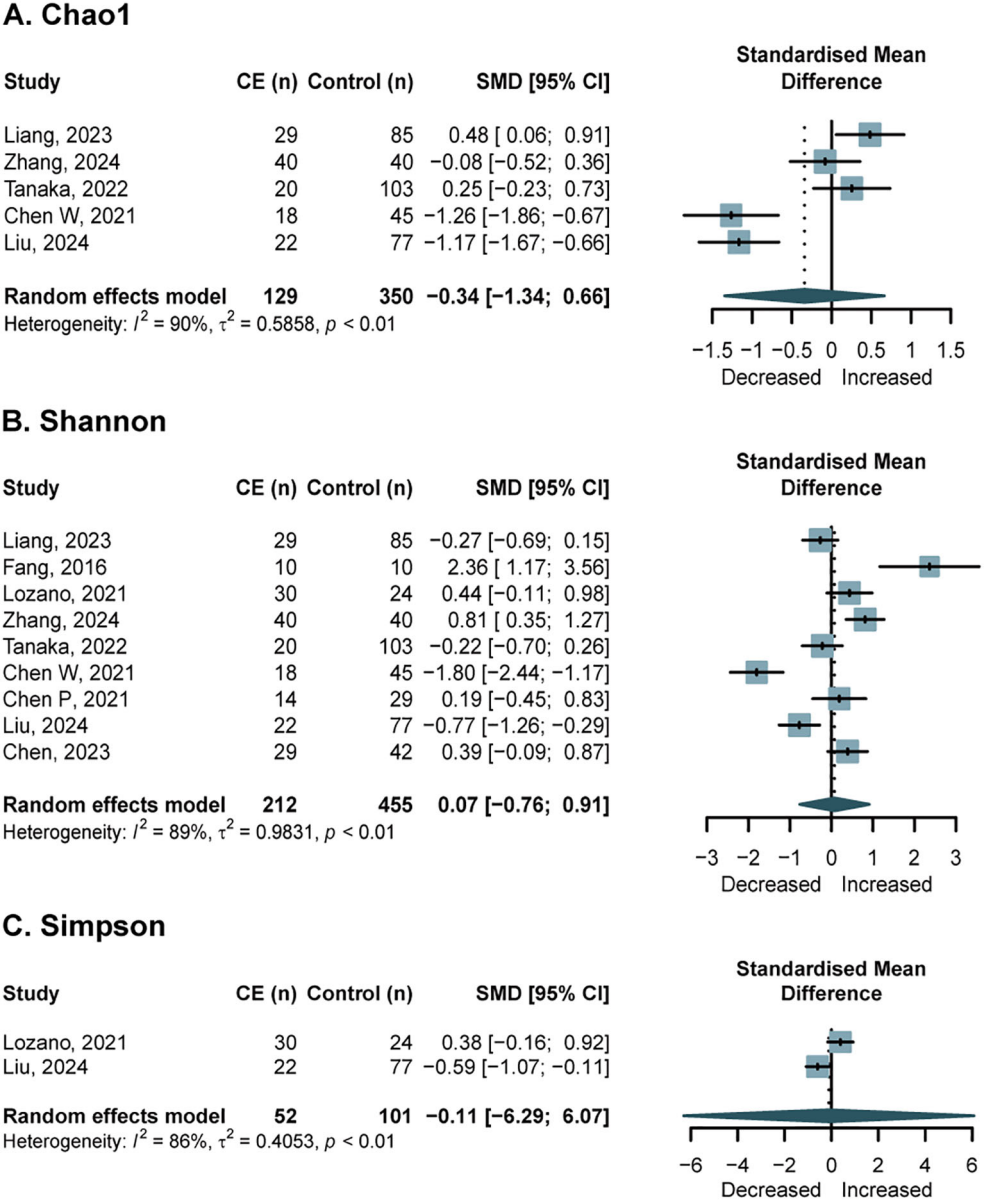
Forest plots of alpha-diversity in the endometrial microbiota of patients with CE compared with NCE controls. **(A)** Chao1; **(B)** Shannon index; **(C)** Simpson index.

Regarding the endometrial microbiota, a meta-analysis of five studies (Chen et al., 2021; Liang et al., 2023; Liu et al., 2024; Tanaka et al., 2022; Zhang et al., 2024) reporting on Chao1 indices (n = 129 patients; n = 350 controls) revealed no significant overall difference between groups (SMD = -0.34, 95% CI, -1.34 to 0.66; *I*^2^ = 59%, *p* = 0.06) (**Figure 3A**). Subgroup analyses and meta-regressions were performed to investigate heterogeneity. No significant associations were found with patient source, sample type, menstrual cycle phase, age, or diagnostic criteria. However, a sub-analysis stratified by the 16S rRNA region showed a significantly elevated Chao1 richness in CE patients in the two studies (Chen et al., 2021; Tanaka et al., 2022) that used the V4 hypervariable region (SMD = 0.38, 95% CI: 0.06 to 0.70) (**Supplementary Table S2**). The stability of these effect estimates was confirmed by a sensitivity analysis that removed low-quality studies (**Supplementary Figure S1A**). Nine studies (Chen et al., 2021, 2023, 2021; Fang et al., 2016; Liang et al., 2023; Liu et al., 2024; Lozano et al., 2021; Tanaka et al., 2022; Zhang et al., 2024) reported Shannon indices (n = 212 patients; n = 455 controls), showing no significant difference between groups (SMD = 0.07; 95% CI, -0.76 to 0.91; *I*^2^ = 89%, *p* < 0.01) (**Figure 3B**). Subgroup analyses and meta-regressions for Shannon indices also yielded no significant associations (**Supplementary Table S3**). A sensitivity analysis, after removing low-quality studies (n = 158 patients; n = 392 controls), similarly found no statistically significant difference (**Supplementary Figure S1B**). Simpson index data were reported by only two studies (n = 52 patients; n = 101 controls) (Liu et al., 2024; Lozano et al., 2021), with a non-significant difference observed between groups (SMD = -0.11; 95% CI, -6.29 to 6.07; *I*^2^ = 86%, *p* < 0.01) (**Figure 3C**). A single study provided phylogenetic diversity data (n = 20 patients; n = 103 controls), revealing no significant intergroup difference (Tanaka et al., 2022), while another study reported a significant decrease in the Ace index in the case group (Liu et al., 2024). Funnel plots for publication bias in Chao1 and Shannon indices are presented in **Supplementary Figure S2**.

### Beta-diversity

Eight studies provided beta-diversity results (Chen et al., 2023; Fang et al., 2016; Han et al., 2024; Liang et al., 2023; Liu et al., 2024; Lozano et al., 2021; Tanaka et al., 2022; Zhang et al., 2024). Among the included studies, four studies (Chen et al., 2023; Liang et al., 2023; Lozano et al., 2021; Tanaka et al., 2022) on endometrial microbiota and two studies (Han et al., 2024; Lozano et al., 2021) on vaginal microbiota consistently found nonsignificant differences. Three studies (Fang et al., 2016; Liu et al., 2024; Zhang et al., 2024), utilizing different assessments, revealed significant differences in β-diversity between patients with CE and controls (**Supplementary Table S4**).

### Differential abundance of microbial taxa

**Figure 4:**
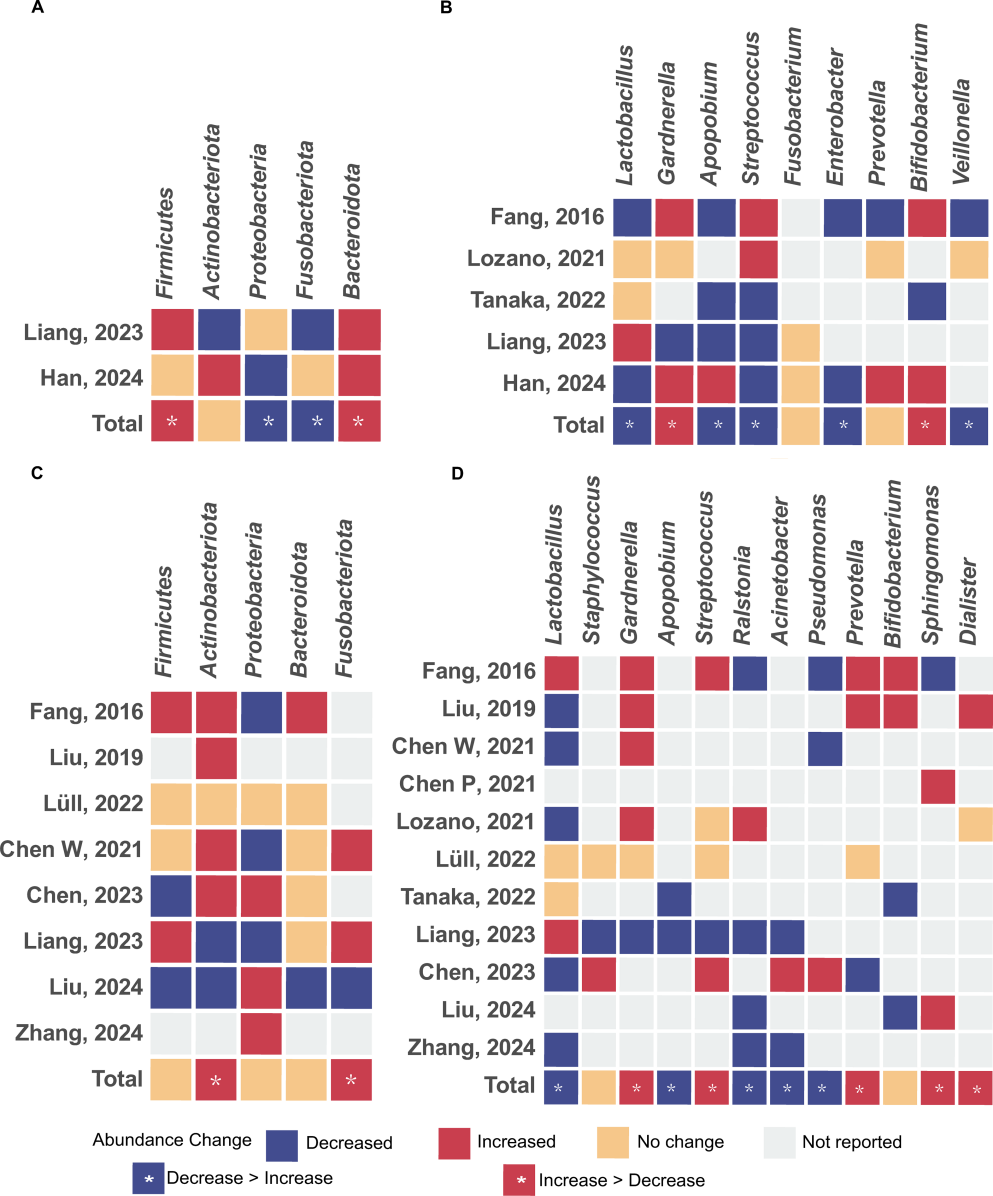
Changes in relative abundance of microbial taxa reported by at least 2 studies. **(A)** Phylum level in vagina; **(B)** Genus level in vagina; **(C)** Phylum level in uterus; **(D)** Genus level in uterus.

Five studies (Fang et al., 2016; Han et al., 2024; Liang et al., 2023; Lozano et al., 2021; Tanaka et al., 2022) reported the relative abundance of vaginal microbiota in CE patients versus NCE controls at the phylum (**Figure 4A**) and genus levels (**Figure 4B**). Except for the increase of *Bacteroidetes* in the CE group, results for other phyla were inconsistent across studies. We observed increased *Gardnerella* and *Bifidobacterium* at the genus level in CE patients when compared to NCE controls. In contrast, *Lactobacillus*, *Apopobium*, *Streptococcus*, *Enterobacter*, and *Veillonella* decreased in CE patients. Notably, the genera with strong support for a decrease in CE cohorts included *Apopobium* (three studies reported the decrease, while one study reported the increase) and *Enterobacter* (two studies).

Ten studies (Chen et al., 2021, 2023, 2021; Fang et al., 2016; Liang et al., 2023; Liu et al., 2024, 2019; Lozano et al., 2021; Lüll et al., 2022; Tanaka et al., 2022; Zhang et al., 2024) reported on endometrial microbiota abundance. Differences spanning five phyla (**Figure 4C**) and twelve genera (**Figure 4D**) were observed. *Actinobacteriota* and *Fusobacteriota* at the phylum level, *Gardnerella*, *Streptococcus*, *Prevotella*, *Sphingomonas*, and *Dialister* at the genus level increased in the CE group. *Lactobacillus*, *Apopobium*, *Ralstonia*, *Acinetobacter*, and *Pseudomonas* at the genus level decreased in CE patients when compared to NCE controls. Among them, *Apopobium* (two studies), *Lactobacillus* (five studies reported the decrease, while one study reported the increase), and *Ralstonia* (four studies reported the decrease, while one study reported the increase) were strongly decreased. *Gardnerella* (four studies reported the increase, one study reported the decrease, and one study found no difference) was strongly increased. Additionally, two studies (Chen et al., 2021; Liu et al., 2024) identified *Sphingomonas* as a significantly enriched genus in CE groups through Linear discriminant analysis.

**Figure 5:**
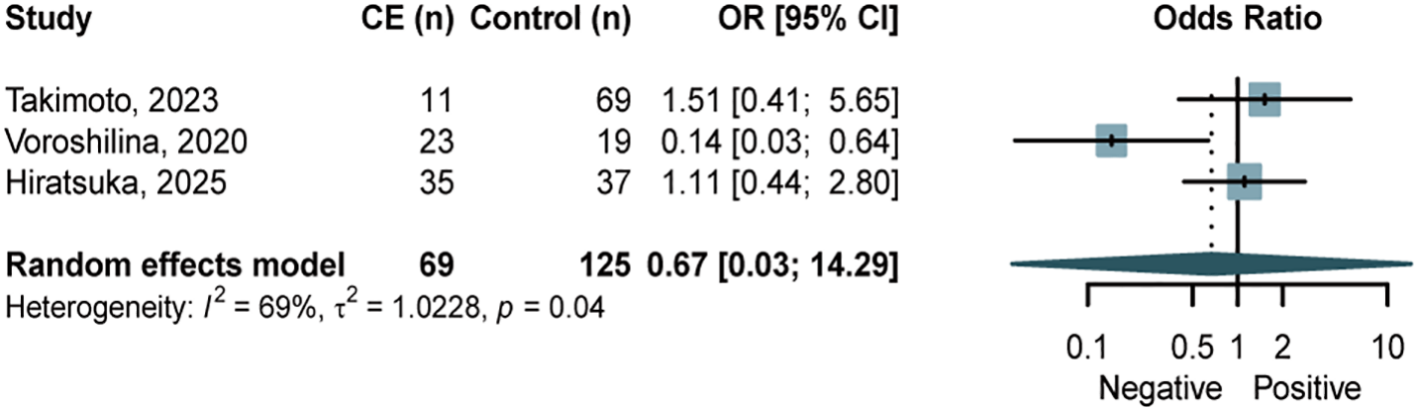
Forest plots assessing the proportion of LD communities between women with CE and NCE controls. LD, Lactobacillus-dominant.

Given that *Lactobacillus* was the most abundant genus in the endometrial microbiota (Chen et al., 2017), we specifically summarized its distribution. Three studies (Hiratsuka et al., 2025; Takimoto et al., 2023; Voroshilina et al., 2020) categorized the endometrial microbiota into *Lactobacillus*-dominant (LD, ≥ 90% *Lactobacillus*) and non-*Lactobacillus*-dominant (NLD) communities, and compared the proportion of LD communities between CE and NCE groups. Pooled analysis revealed no significant intergroup differences (**Figure 5**). Funnel plots for publication bias is presented in **Supplementary Figure S3**.

### Microbial detection rates in CE

**Figure 6:**
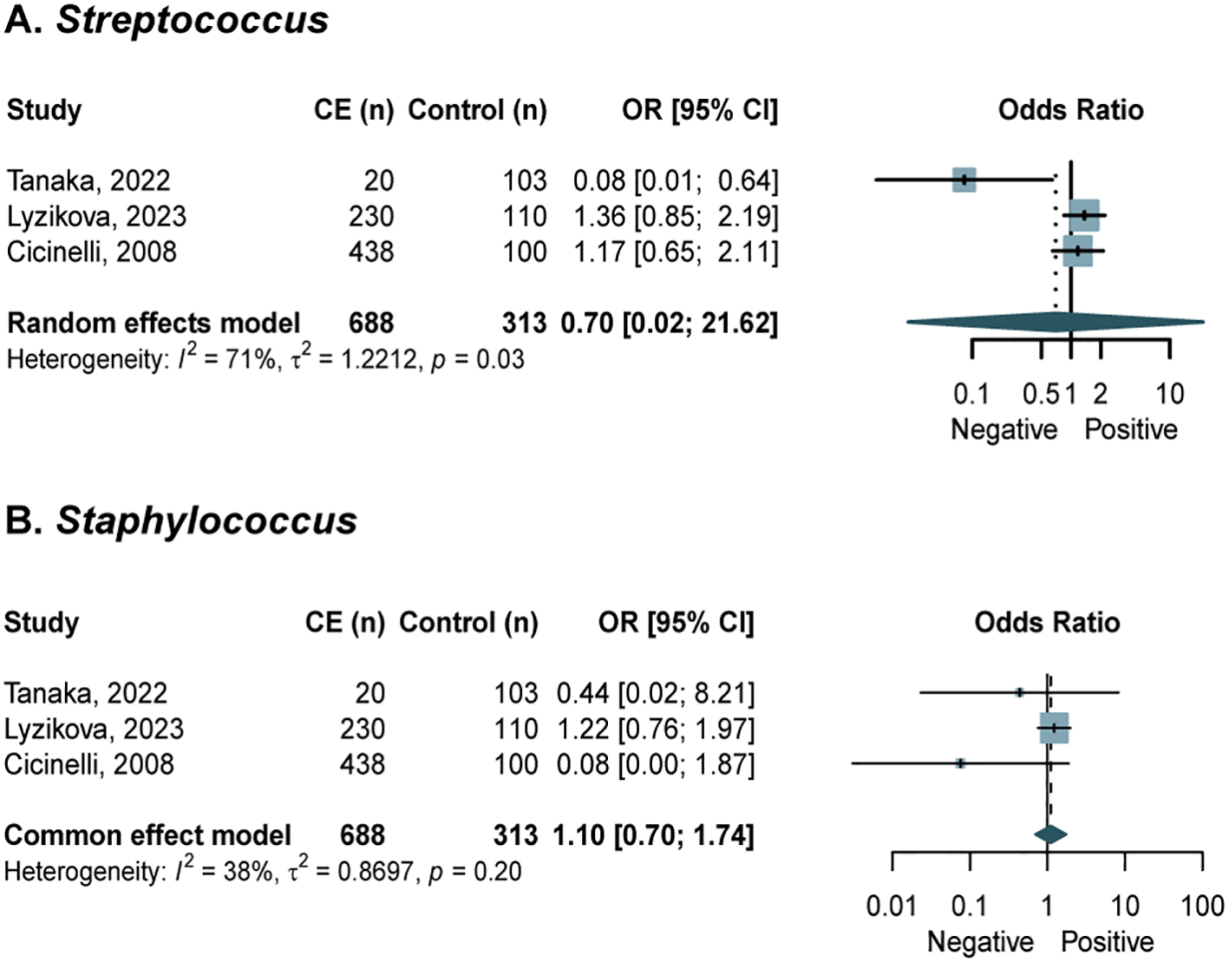
Forest plots assessing the differences in microbial detection rate of vaginal microbiota between women with CE and NCE controls. **(A)** Streptoccus; **(B)** Staphylococcus.

**Figure 7:**
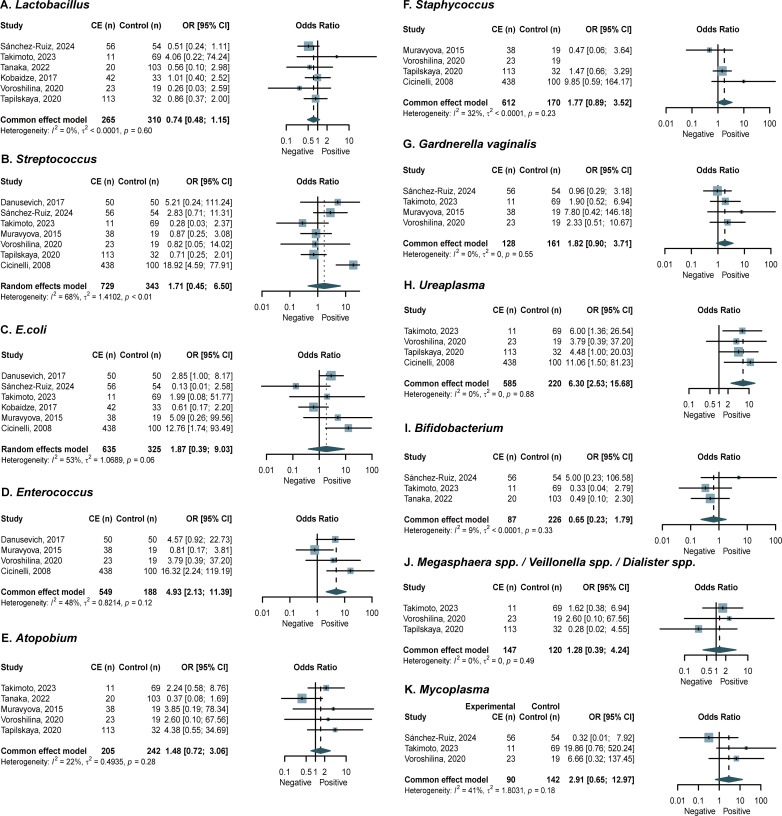
Forest plots assessing the differences in microbial detection rate of endometrial microbiota between women with CE and NCE controls. **(A)** Lactobacillus; **(B)** Streptococcus; ; **(C)** E. coli **(D)** Enterococcus; **(E)** Atopobium; **(F)** Staphycoccus; **(G)** Gardnerella; **(H)** Ureaplasma; **(I)** Bifidobacterium; **(J)** Megasphaera spp./Veillonella spp./Dialister spp.; **(K)** Mycoplasma.

A meta-analysis of microbial detection rates only for specific agents reported in three or more studies. Our analysis revealed comparable detection rates of *Streptococcus* and *Staphylococcus* in vaginal samples between CE patients and control groups (**Figure 6**) (Cicinelli et al., 2008; Lyzikova, 2023; Tanaka et al., 2022). Pooled analysis of endometrial microbiota revealed consistent detection rates of *Lactobacillus*, *E. coli*, *Streptococcus*, *Staphycoccus*, *Atopobium*, *Gardnerella*, *Bifidobacterium*, *Megasphaera* spp./*Veillonella* spp./*Dialister* spp., and *Mycoplasma* in CE versus controls, with significantly higher prevalence rates observed for *Enterococcus* (OR = 4.93; 95% CI, 2.13 to 11.39; *I*^2^ = 48%, *p* = 0.12) and *Ureaplasma* (OR = 6.30; 95% CI, 2.53 to 15.68; *I*^2^ = 0%, *p* = 0.88) in the CE group (**Figure 7**) (Cicinelli et al., 2008; Danusevich et al., 2017; Muravyova et al., 2015; Sánchez-Ruiz et al., 2025; Takimoto et al., 2023; Tanaka et al., 2022; Tapilskaya et al., 2020; Voroshilina et al., 2020). Subgroup analysis and meta-regressions (endometrial *Streptococcus*) revealed that the method of assessment and diagnostic criteria between the CE and control groups did not show any significant associations (**Supplementary Table S5**). Sensitivity analysis performed by removing low-quality studies confirmed the stability of effect estimates (**Supplementary Figure S1C**). The funnel plots of publication bias in the detection rate were presented in **Supplementary Figure S3**.

## Discussion

This systematic review and meta-analysis systematically compared the vaginal and endometrial microbiota composition between CE patients and controls, identifying key microbial alterations significantly associated with CE. The findings are summarized as follows: (1) alpha-diversity indices (Chao1/Shannon/Simpson) showed no statistically significant intergroup variations; (2) although some studies reported differences in vaginal and endometrial microbiota composition between the groups, findings regarding beta-diversity were inconsistent across studies; (3) *Lactobacillus* predominated in both vaginal and endometrial microbiota, yet its abundance was significantly reduced in CE, whereas pro-inflammatory microorganisms were consistently upregulated.

Microbial communities exist as a continuum across the female reproductive tract, changing from the vagina to the ovaries. While vaginal *Lactobacillus* species inhibit the growth of other microbes, a healthy uterine microbiota may also be influenced by the uterine nutrients and hormones, along with the microbiota in vagina and the peritoneal cavity. Therefore, both vaginal and endometrial flora are essential for identifying microorganisms associated with CE patients.

Alpha-diversity serves as the ecological index for characterizing microbial community complexity, including richness (e.g., Chao1) and evenness (e.g., Shannon, Simpson). There were variances across all studies, with no statistically significant differences observed when the Chao1, Shannon, and Simpson indices were assessed. This suggests that the richness and evenness of the reproductive tract microbiota in patients with CE may not exhibit significant alterations. Given the potential confounding effects of menstrual cycle phase, sample type, and 16S rRNA sequencing region on reproductive tract microbiota composition (Chen et al., 2017; Liu et al., 2024; Lüll et al., 2022), we performed subgroup analyses. Only the endometrial Chao1 index showed a significant increase, specifically in studies utilizing the V4 hypervariable region for sequencing. Compared to the V3-V4 hypervariable region, sequencing relying on the V4 region may be subject to limitations in resolution. In addition, subgroup analyses of other diversity indices still showed no significant differences between the two groups, which may also be attributed to the limited number of studies. Regarding beta-diversity, the findings remained inconsistent across studies. Three investigations (Fang et al., 2016; Liu et al., 2024; Zhang et al., 2024) identified distinct clustering patterns between the microbial communities of CE patients and non-CE controls. Further research is needed to elucidate the relationships between vaginal and endometrial microbiota (including both alpha- and beta-diversity) and CE.

Although the microbiota showed preserved alpha-diversity, subtle microbial compositional changes may exist. *Lactobacillus* is the most abundant genus in both vaginal and endometrial microbial communities (Chen et al., 2017). The observed decrease in *Lactobacillus* abundance in both vaginal and endometrial microbiota is a critical finding. *Lactobacillus* provides critical defense against pathogenic invasion through the production of lactic acid and hydrogen peroxide, its depletion in CE patients may facilitate ascending infection by pathogenic microorganisms (Zhu et al., 2022). Previous studies suggest that *Lactobacillus*-dominated microbiota may benefit embryo implantation (Moreno et al., 2016). Our meta-analysis found no significant association between *Lactobacillus* dominance (≥ 90% abundance) and CE, possibly due to this stringent threshold, since endometrial *Lactobacillus* typically constitutes 30.6% in healthy women (Chen et al., 2017). Future studies should establish more appropriate thresholds. While the balance between *L. crispatus* and *L. iners* may also influence disease progression (Koedooder et al., 2019; Zhu et al., 2022), this meta-analysis could not aggregate such species-specific data due to limitations in the resolution of 16S amplicon sequencing and few studies reporting at the species level.

*Gardnerella*, belonging to the phylum *Actinobacteria*, shows low abundance in the endometrium. The increase in *Gardnerella*, coupled with decreased *Lactobacillus*, resembles the microbial profile of bacterial vaginosis (Swidsinski et al., 2023). Several studies also reported a higher prevalence of prior vaginal infections among CE patients (Haggerty et al., 2004; Ravel et al., 2021). These findings suggest an increased likelihood of ascending infection. Furthermore, the amino acids generated by *Gardnerella* can be utilized mutually by *Prevotella* species and may foster the growth of other bacteria (Shvartsman et al., 2023).

Two independent studies (Chen et al., 2021; Liu et al., 2024) utilizing linear discriminant analysis (LDA) consistently identified *Sphingomonas* as a significantly enriched genus in CE patients compared to controls. *Sphingomonas* was mainly positively related to dendritic cells, natural killer cells, induced regulatory T cells, and B cells (Chen et al., 2021). This finding aligns with the observed increase in the endometrium of CE patients (Li et al., 2020), suggesting that the microbial shift is not merely a sign of infection, but a potent modulator of the local immune landscape.

In addition, the markedly higher prevalence of *Enterococcus* and *Ureaplasma* in CE patients strongly implicates these microorganisms in disease pathogenesis. *Enterococcus* may potentially utilize biofilm formation as a virulence factor and cause a decline in the population of *Lactobacillus* (Sengupta et al., 2021). *Ureaplasma* species are frequently found colonizing the adult genitourinary tract and considered low-virulence commensals. *Ureaplasma* is increasingly recognized as an opportunistic pathogen in human genitourinary tract infections, infertility, adverse pregnancy outcomes, neonatal morbidities, and so on (Liu et al., 2025). The colonization of *Ureaplasma* may lead to elevated levels of pro-inflammatory cytokines such as IL-6 (Sprong et al., 2020), and promote an immune-tolerant microenvironment, potentially facilitate its long-term colonization, and contribute to chronic infection (Teixeira Oliveira et al., 2021).

Meanwhile, the observed microbial shifts indicate an imbalance in short-chain fatty acids (SCFAs) in the microenvironment. The decline in lactate-producing taxa coupled with the rise in acetate and succinate-producing pathobionts may disrupt mucosal acidification, promoting a pro-inflammatory state (Meng et al., 2024). These collective changes likely contribute to barrier dysfunction and immune dysregulation.

Recent studies have suggested that the gut microbiota may also influence uterine pathophysiology and inflammation (Hagihara et al., 2024; Iavarone et al., 2023; Qiu et al., 2025). Our findings revealed a reduction of *Lactobacillus*, with an expansion of vaginal *Bifidobacterium* and endometrial *Prevotella* and *Streptococcus*. Notably, this microbial signature parallels the gut dysbiosis observed in patients with endometriosis (Iavarone et al., 2023). Dysbiosis of the gut microbiota can trigger impaired the intestinal barrier, metabolic perturbations and elevated pro-inflammatory cytokine, which could subsequently modulate the local uterine microenvironment. Given the significant association between endometriosis and CE (Kalaitzopoulos et al., 2025), these findings suggest that microbial dysbiosis in both the reproductive and gastrointestinal tracts may contribute to the pathogenesis of CE in a similar manner.

CE is significant related to RIF, RPL, and infertility (Bouet et al., 2016; Liu et al., 2018; McQueen et al., 2015). The microbiota of the reproductive tract plays a crucial role in embryo implantation (Benner et al., 2018). Previous studies have found that microbial shifts in the reproductive tract, such as a decrease in *Lactobacillus* and an increase in *Enterococcus*, *Streptococcus*, *Sphingomonas*, and other unfavorable microorganisms, may contribute to infertility and embryo implantation failure (Chen et al., 2022, 2025; Fu et al., 2020). Our data suggest that certain abnormal microorganisms might be one of the factors related to pregnancy failure in CE patients. These findings may provide a clinical reference for evaluating why some patients experience adverse pregnancy outcomes. Furthermore, this data could provide some evidence for identifying patients at high risk of adverse reproductive outcomes and potentially supporting targeted strategies to improve pregnancy rates in the future.

Our findings, demonstrate a consistent and distinct microbial compositional shift in the reproductive tract of CE patients, which is not reflected by an overall change in microbial richness or evenness. This suggests that the dysbiosis in CE is not a matter of quantitative complexity, characterized by the replacement of beneficial commensals with opportunistic pathogens.

The stronger microbial associations in endometrial samples suggest that local, tissue-specific microbiota-immune interactions may be particularly relevant to disease development. The microbial differences between chronic endometritis patients and controls vary between the endometrium and vagina, suggesting selective colonization in the uterine cavity rather than simple ascending infection from the vagina. This spatial specificity highlights the necessity of endometrial sampling for accurate CE diagnosis and underscores why vaginal swabs may be insufficient.

Our study exhibits several distinct strengths. First, the meta-analysis significantly expands the total sample size by aggregating data, which provides robust statistical power. Second, this is the first systematic review and meta-analysis to simultaneously synthesize both vaginal and endometrial microbial alterations in patients with CE. This provides a more comprehensive understanding of the reproductive tract’s microbial landscape than studies confined to a single site. Third, we performed subgroup analyses to address methodological heterogeneity, allowing for a more accurate interpretation of microbial alterations. Furthermore, this study establishes a solid foundation for the future identification of microbial biomarkers associated with CE and emphasizes the clinical necessity of endometrial sampling.

Despite the significant findings, the current analysis has several limitations, including the predominance of small-scale studies and the considerable methodological heterogeneity, particularly inconsistent diagnostic criteria for CE and microbiome assessment methods. The sampling methods varied across the included studies (e.g., endometrial fluid aspiration versus tissue biopsy), which may influence the specific microbial profiles (Lüll et al., 2022). Additionally, potential confounding factors such as medical history were not fully adjusted for in some studies. Some included studies did not clearly define the required period of antibiotic non-use. The lack of a standardized washout period may have introduced potential confounding factors into the analysis. Importantly, microbial profiles in patients with endometriosis are different (Salliss et al., 2021), and CE is closely related to endometriosis (Kalaitzopoulos et al., 2025). However, these factors were not adequately considered during subject inclusion and exclusion in the original studies. These factors collectively highlight the need for cautious interpretation of the findings.

Future research must address these limitations by adopting standardized, large-scale, multicenter study designs. Furthermore, multi-omics integration (microbiomics, metabolomics, transcriptomics, and so on) are needed to fully elucidate the complex functional interactions between the dysbiotic microbial community and the endometrial immune microenvironment. This deeper understanding will be essential for the development of targeted, pathogen-directed therapies that move beyond broad-spectrum antibiotics and potentially incorporate personalized probiotic strategies based on the unique microbiome profile.

## Conclusions

This systematic review and meta-analysis provide strong evidence for a microbial basis of CE. The lack of significant changes in overall microbial diversity is coupled with a distinct shift in the composition of the vaginal and endometrial microbiota. This shift is marked by a decrease in *Lactobacillus* and a significant increase in the prevalence of key pathogens like *Gardnerella* in vagina and uterus, *Sphingomonas*, *Enterococcus* and *Ureaplasma* in uterus, highlighting a pro-inflammatory microbial state. These findings extended the results from previous studies and underscore the critical role of the microbial-immune crosstalk in CE pathogenesis. While limited study numbers and methodological heterogeneity warrants cautious interpretation and further research for validation, our results provide evidence for future etiological exploration and the development of therapeutic strategies for this challenging condition.

## Data Availability

The original contributions presented in the study are included in the article/**Supplementary Material**. Further inquiries can be directed to the corresponding author.
