# Supplementary material for: Vaginal and endometrial microbiota dysbiosis in patients with chronic endometritis: a systematic review and meta-analysis
Source: Front Cell Infect Microbiol. 2026 Feb 20;16:1754297. doi: 10.3389/fcimb.2026.1754297 (PMC12963329; doi:10.3389/fcimb.2026.1754297)
Supplement: Supplementary file 1 [file DataSheet1.docx]

**Supplementary material**

Table S1. Quality assessment of the included original studies.

Table S2. Subgroup and meta-regression analysis of endometrial Chao1 indices difference between CE patients and controls.

Table S3. Subgroup and meta-regression analysis of endometrial Shannon indices difference between CE patients and controls.

Table S4. Methodology and findings of the included studies assessing beta diversity.

Table S5. Subgroup and meta-regression analysis of endometrial *Streptococcus* between CE patients and controls.

Figure S1 Sensitivity analyses for the Chao1, the Shannon index and *Streptococcus* detection rate in uterus.

Figure S2. Funnel plots assessing publication bias in the meta-analyses of alpha-diversity.

Figure S3. Funnel plots assessing publication bias in the meta-analyses of microbial detection rate and the proportion of *Lactobacillus*-dominant communities.

Table S1. Quality assessment of the included original studies.

| **Study** | **1.** **Were the groups comparable other than the presence of disease in cases or the absence of disease in controls?** | **2. Were cases and controls matched appropriately?** | **3. Were the same criteria used for identification of cases and controls?** | **4. Was exposure measured in a standard, valid and reliable way?** | **5. Was exposure measured in the same way for cases and controls?** | **6. Were confounding factors identified?** | **7. Were strategies to deal with confounding factors stated?** | **8. Were outcomes assessed in a standard, valid and reliable way for cases and controls?** | **9. Was the exposure period of interest long enough to be meaningful?** | **10. Was appropriate statistical analysis used?** | **Risk** |
| --- | --- | --- | --- | --- | --- | --- | --- | --- | --- | --- | --- |
| Cicinelli, 2008 | Y | Y | Y | Y | Y | Y | Y | Y | Y | Y | L |
| Chen, 2021a | Y | Y | Y | Y | Y | Y | Y | Y | Y | Y | L |
| Chen, 2021b | Y | Y | Y | Y | Y | N | N | Y | Y | Y | H |
| Chen, 2023 | Y | Y | Y | Y | Y | Y | Y | Y | Y | Y | L |
| Danusevich, 2017 | Y | Y | Y | Y | Y | Y | N | Y | Y | Y | H |
| Fang, 2016 | N | Y | N | Y | Y | N | N | Y | Y | Y | H |
| Han, 2024 | Y | Y | Y | Y | Y | Y | Y | Y | Y | Y | L |
| Hiratsuka, 2025 | Y | Y | Y | Y | Y | Y | Y | Y | Y | Y | L |
| Kobaidze, 2017 | N | N | N | Y | Y | N | N | Y | Y | Y | H |
| Liang, 2023 | Y | Y | Y | Y | Y | Y | Y | Y | Y | Y | L |
| Liu, 2019 | Y | Y | Y | Y | Y | Y | Y | Y | Y | Y | L |
| Liu, 2024 | Y | Y | Y | Y | Y | Y | Y | Y | Y | Y | L |
| Lozano, 2021 | Y | Y | Y | Y | Y | N | N | Y | Y | Y | H |
| Lüll. 2022 | Y | Y | Y | Y | Y | N | N | Y | Y | Y | H |
| Lyzikova, 2023 | Y | Y | Y | Y | Y | Y | N | Y | Y | Y | H |
| Muravyova, 2015 | Y | Y | Y | Y | Y | N | N | Y | Y | Y | H |
| Sánchez-Ruiz, 2024 | Y | Y | Y | Y | Y | Y | Y | Y | Y | Y | L |
| Tanaka, 2022 | Y | Y | Y | Y | Y | Y | Y | Y | Y | Y | L |
| Takimoto, 2023 | Y | Y | Y | Y | Y | Y | Y | Y | Y | Y | L |
| Tapilskaya, 2020 | Y | Y | Y | Y | Y | Y | Y | Y | Y | Y | L |
| Voroshilina, 2020 | Y | Y | Y | Y | Y | N | N | Y | Y | Y | H |
| Zhang, 2024 | Y | Y | Y | Y | Y | Y | Y | Y | Y | Y | L |

Joanna Briggs Institute Critical Appraisal Checklist for Case Control Studies; Y, yes; N, no; H, high-risk; L, low-risk.

Table S2. Subgroup and meta-regression analysis of endometrial Chao1 indices difference between CE patients and controls.

| **Sub-group** | **N** | **SMD (95%CI)** | **I^2^** | **Model** | **Meta-regression OR, (95%CI), p-value** |  |
| --- | --- | --- | --- | --- | --- | --- |
| **Sample type** | | | | | | |
| ET | 3 | -0.63 (-3.09, 1.82) | 94.0% | Random | 0.49, (0.05, 5.21), 0.41 |  |
| EF | 2 | 0.07 (-0.25, 0.39) | 2.3% | Fixed | 1 |  |
| **Diagnostic criteria** | | | | | | |
| Weakly | 2 | -0.37 (-11.46,10.73) | 95.5% | Random | 0.96, (0.06, 14.52), 0.97 |  |
| Strongly | 3 | -0.33 (-2.17, 1.51) | 88.6% | Random | 1 |  |
| **Patient source** | | | | | | |
| Infertility | 3 | -0.14 (-2.35, 2.08) | 92.5% | Random | 1 |  |
| RIF | 2 | -0.66 (-8.16, 6.85) | 89.9% | Random | 0.59, (0.05, 7.56), 0.56 |  |
| **Phase of the menstrual cycle** | | | | | | |
| Proliferative | 1 | - | - | - | 1 |  |
| Secretory | 3 | -0.35 (-2.31, 1.61) | 87.5% | Random | 0.44, (0.01, 20.94), 0.45 |  |
| **Age** | | | | | | |
| >=35 | 1 | - | - | - | 1 |  |
| <35 | 3 | -0.25(--2.32, 1.83) | 91.8% | Random | 0.61, (0.01, 39.02), 0.66 |  |
| **16S region** | | | | | | |
| V4 | 2 | 0.38 (0.06, 0.70) | 0% | Fixed | 3.27, (0.67, 15.80), 0.10 |  |
| V3-V4 | 3 | -0.81 (-2.46, 0.84) | 86.1% | Random | 1 |  |

Table S3. Subgroup and meta-regression analysis of endometrial Shannon indices difference between CE patients and controls.

| **Sub-group** | **N** | **SMD (95%CI)** | **I^2^** | **Model** | **Meta-regression OR, (95%CI), p-value** |
| --- | --- | --- | --- | --- | --- |
| **Sample type** | | | | | |
| ET | 5 | 0.30 (-1.10, 1.71) | 86.1% | Random | 1.63, (0.27, 9.83), 0.54 |
| EF | 4 | -0.19 (-2.00, 1.62) | 93.5% | Random | 1 |
| **Diagnostic criteria** | | | | | |
| Weakly | 3 | 0.12 (-0.78,1.01) | 53.6% | Random | 1.05, (0.15, 7.38), 0.95 |
| Strongly | 6 | -0.06 (-1.38, 1.50) | 92.9% | Random | 1 |
| **Patient source** | | | | | |
| Infertility | 6 | -0.36 (-1.22, 0.50) | 87.6% | Random | 1 |
| RIF | 2 | 0.51 (-3.43, 4.46) | 58.4% | Random | 2.39, (0.51, 11.21), 0.22 |
| **Phase of the menstrual cycle** | | | | | |
| Proliferative | 3 | 0.71 (-2.56, 3.97) | 88.8% | Random | 1 |
| Secretory | 5 | -0.11 (-1.36, 1.15) | 91.4% | Random | 0.44, (0.06, 3.31), 0.36 |
| **Age** | | | | | |
| >=35 | 2 | 0.11 (-4.06, 4.27) | 93.0% | Random | 1 |
| <35 | 6 | 0.06 (-1.38, 1.51) | 68.2% | Random | 0.96, (0.08, 11.90), 0.97 |
| **16S region** | | | | | |
| V4 | 5 | -0.01 (-1.77, 1.79) | 91.7% | Random | 0.85, (0.13, 5.41), 0.85 |
| V3-V4 | 4 | 0.17 (-0.91, 1.25) | 86.9% | Random | 1 |

Table S4. Methodology and findings of the included studies assessing beta diversity

| **Study** | **Sample type** | **Metric** | **Analysis** | **Finding** |
| --- | --- | --- | --- | --- |
| Chen et al., 2023 | EF | Not reported | PAM | no sig. difference |
| Fang et al., 2016 | ET | weighted UniFrac | PCoA/ ANOSIM | sig. different |
| Han et al., 2024 | VS | Bray-Curti | PCoA/ ANOSIM | no sig. difference |
| Liang et al., 2023 | ET | Not reported | PCoA | no sig. difference |
| Liu et al., 2024 | ET | weighted UniFrac | PCoA/NMDS/ ANOSIM | sig. different |
| Lozano et al., 2021 | ET, VS | Not reported | PCoA | no sig. difference |
| Tanaka et al., 2022 | EF | UniFrac | PCoA | no sig. difference |
| Zhang et al., 2024 | EF | Bray-Curtis | PCoA/Adonis | sig. different |

PAM, partitioning around medoids; PCoA, principal coordinates analysis; ANOSIM, analysis of similarities, Adonis: non-parametric multivariate analysis of variance

Table S5. Subgroup and meta-regression analysis of endometrial *Streptococcus* between CE patients and controls.

| **Sub-group** | **N** | **SMD (95%CI)** | **I^2^** | **Model** | **Meta-regression OR, (95%CI), p-value** |
| --- | --- | --- | --- | --- | --- |
| **Method** | | | | | |
| Culture | 4 | 3.70 (0.42, 32.63) | 70.7% | Random | 12.92, (0.22, 750.55), 0.16 |
| PCR | 2 | 0.73 (0.25, 2.01) | 0% | Fixed | 2.63, (0.03, 217.96), 0.58 |
| 16S | 1 | 0.28 (0.03, 2.37) | - | - | 1 |
| **Diagnostic criteria** | | | | |  |
| Weakly | 5 | 3.03 (0.56, 16.46) | 64.0% | Random | 5.99, (0.46, 77.48), 0.13 |
| Strongly | 2 | 0.56 (0.23, 1.37) | 0% | Fixed | 1 |


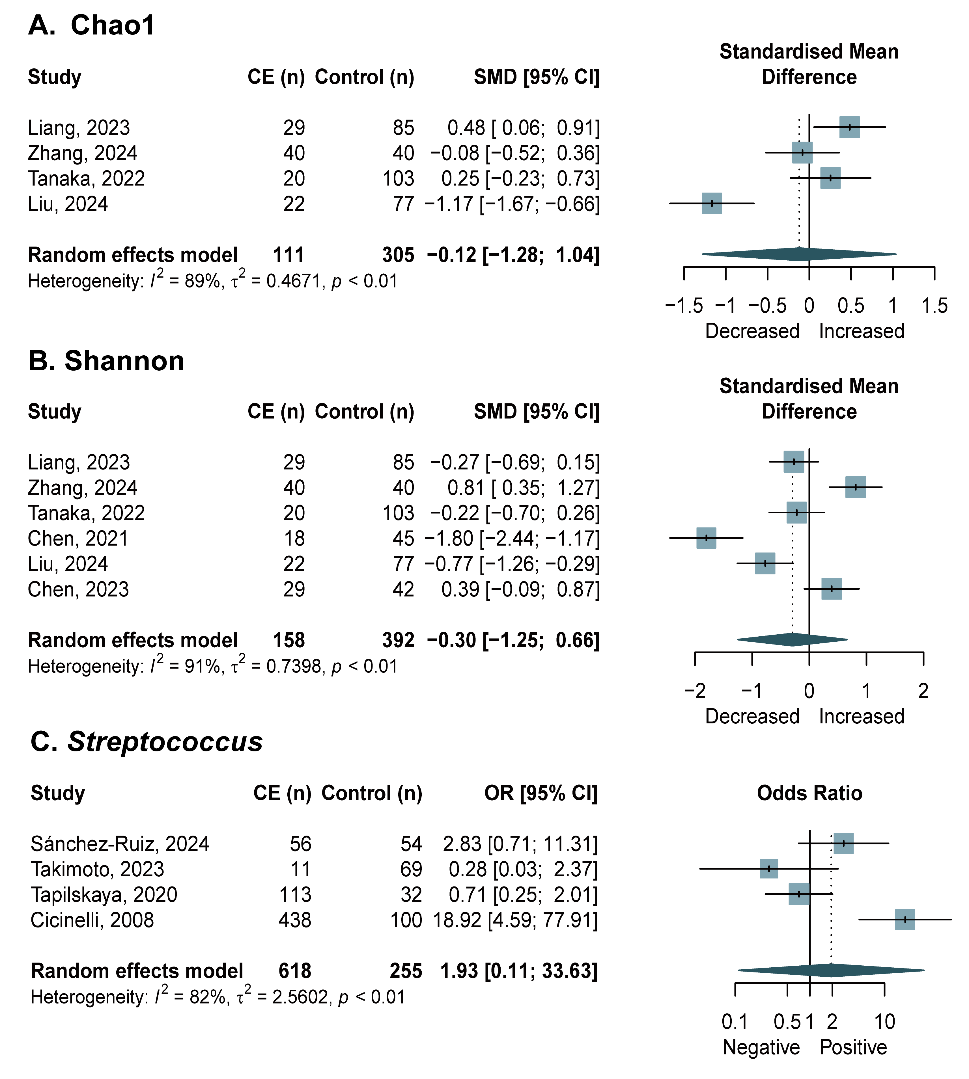


Figure S1 Sensitivity analyses for the Chao1 (A), the Shannon index (B) and *Streptococcus* detection rate (C) in uterus.


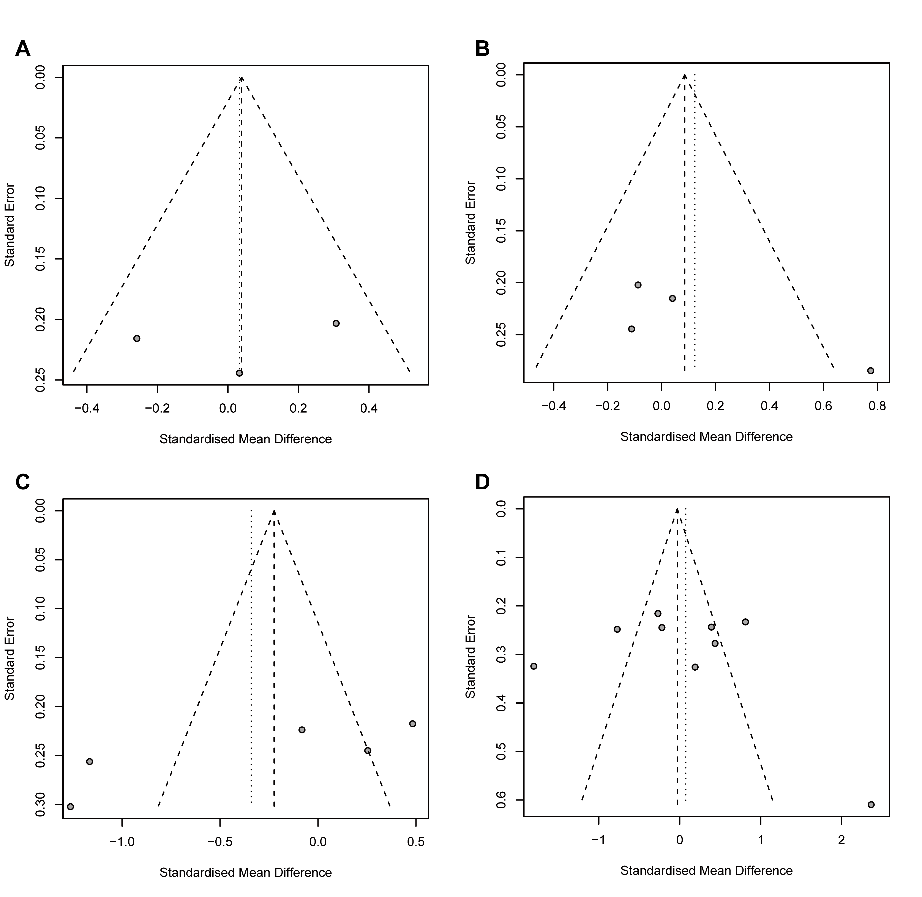


Figure S2. Funnel plots assessing publication bias in the meta-analyses of alpha-diversity. (A-B) Chao1, Shannon in vagina; (C-D) Chao1, Shannon in uterus.


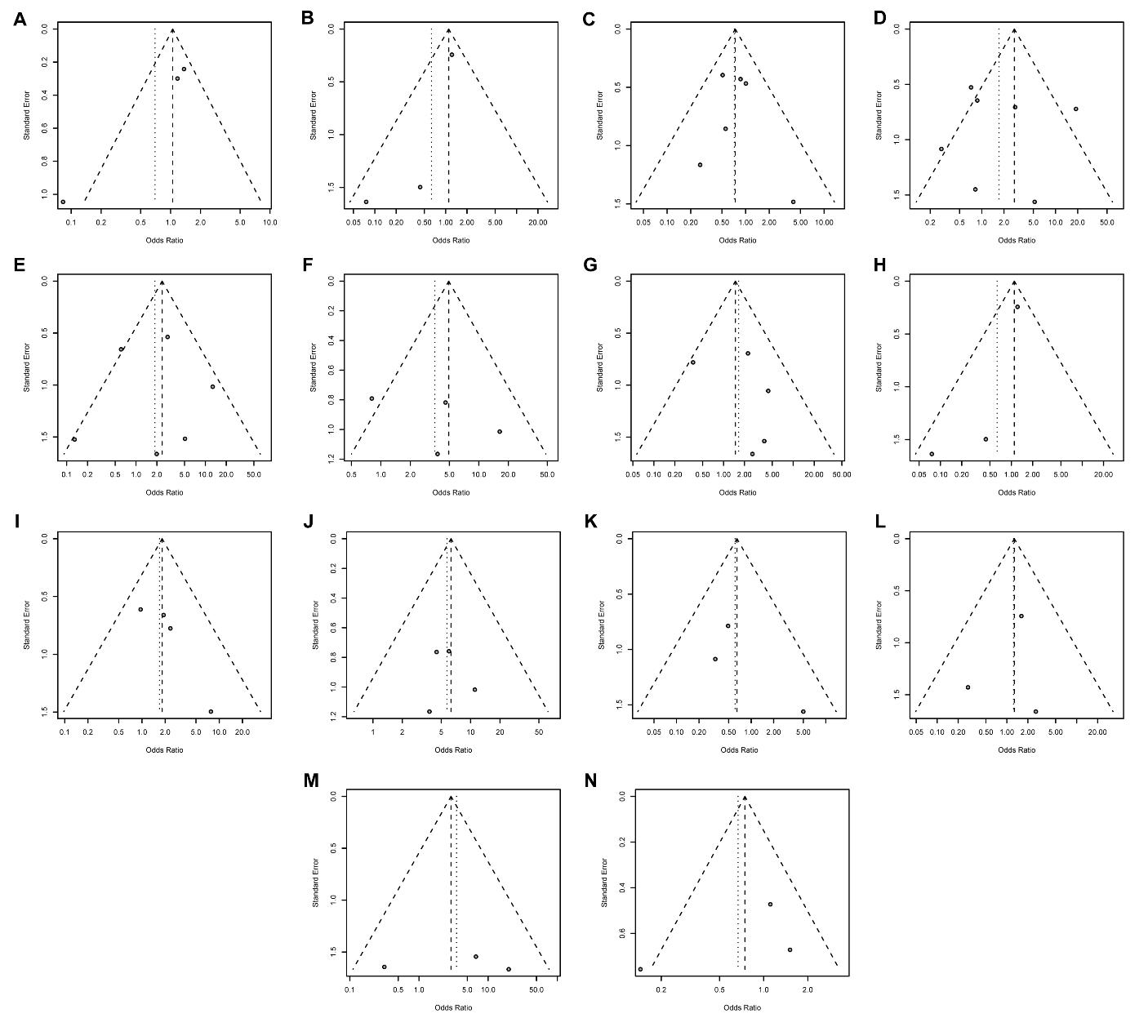


Figure S3. Funnel plots assessing publication bias in the meta-analyses of microbial detection rate and the proportion of *Lactobacillus*-dominant communities. (A-B) *Streptococcus* and *Staphylococcus* in vagina; (C-M) *Lactobacillus*, *Streptococcus*, *E. coli*, *Enterococcus*, *Atopobium*, *Staphycoccus*, *Gardnerella*, *Ureaplasma*, *Bifidobacterium*, *Megasphaera* spp./*Veillonella* spp./*Dialister* spp., and *Mycoplasma* in uterus; (N) the proportion of *Lactobacillus*-dominant communities.
